# Low-Load Blood Flow Restriction Squat as Conditioning Activity Within a Contrast Training Sequence in High-Level Preadolescent Trampoline Gymnasts

**DOI:** 10.3389/fphys.2022.852693

**Published:** 2022-06-13

**Authors:** Shengtao Yang, Peng Zhang, Marta Sevilla-Sanchez, Dong Zhou, Jie Cao, Jiajian He, Binghong Gao, Eduardo Carballeira

**Affiliations:** ^1^ School of Physical Education and Training, Shanghai University of Sport, Shanghai, China; ^2^ Professional Sports Research Center, Shanghai Research Institute of Sports Science, Shanghai, China; ^3^ Department of Physical Education and Sport, Faculty of Sport Sciences and Physical Education, Campus Bastiagueiro, University of A Coruna, Oleiros, Spain

**Keywords:** blood flow restriction training, young athletes, contrast strength training, jump height, maximal isometric strength, high-load resistance exercise

## Abstract

To investigate the effects of implementing low-load blood flow restriction exercises (LL-BFRE) instead of high-load exercises (HL-RE) in a contrast training program on strength and power performance of high-level young gymnasts. Fifteen high-level pre-pubescent trampoline gymnasts (national level, Tanner Stage II, intermediate experience in strength training) were divided into two groups to complete the same structure of a ten-week contrast strength training program differing only in the configuration of the first resistance exercise of the contrast sequence. The LL-BFRE group (*n* = 7, four girls, 13.9 ± 0.4 y) performed the first resistance exercise of the contrast with LL-BFRE (20%–30% 1RM, perceived pressure of 7 on a scale from 0 to 10). The HL-RE group (*n* = 8, four girls, 13.8 ± 0.5 y) trained the first resistance exercise of the contrast sequence with moderate-to-high load (60%–85% 1RM). Before and after the training period, isometric mid-thigh pull (IMTP), squat jump (SJ), counter movement jump (CMJ), and drop-jump (DJ) were performed to evaluate the effect of the intervention on strength and power capacities as primary outcomes. Changes in participants’ anthropometric measures, muscle mass, left and right thigh girth, IMTP relative to bodyweight (IMTP-R), eccentric utilization ratio (EUR), and reactive strength index (RSI) were assessed as secondary outcomes. There was no significant interaction (*p* > 0.05) between group x time in any power and strength outcome, although SJ and EUR showed a trend to significant interaction (*p* = 0.06 and *p* = 0.065, respectively). There was an overall effect of time (*p* < 0.05) in all power and strength variables (CMJ, SJ, EUR, DJ, RSI, IMTP, and IMTP-R). There was a significant interaction in muscle mass (MM) [β = 0.57 kg, 95% CI = (0.15; 0.98), t_13_ = 2.67, *p* = 0.019], revealing that participants in LL-BFRE increased their muscle mass (6.6 ± 3.1%) compared to HL-RE (3.6 ± 2.0%). Anthropometric variables did not present any group or interaction effect. However, there was a time effect (*p* < 0.05). Implementing LL-BFRE in place of HL-RE as a conditioning activity in a contrast training sequence might be equally effective in improving lower-body strength and power in preadolescent trampoline gymnasts.

## 1 Introduction

The trampoline gymnasts need high levels of power in their lower limbs to achieve an optimal height in the initial part of a routine and dispose of more aerial time for the functional part to encompass forwards and backward somersaults and twists (Jensen et al., 2013). Resistance training under close supervision and with correct guidance has been proven effective and beneficial in developing strength and power and enhancing athletic performance in young athletes (Behringer et al., 2010; Granacher et al., 2011; Harries et al., 2012; Granacher et al., 2016; Suchomel et al., 2016; McQuilliam et al., 2020). In general, it has been suggested that lifting high-load resistances (i.e., ≥80% of 1 repetition maximum, 1RM) increases strength and power in young athletes with advanced or intermediate experience (Faigenbaum et al., 2009; Lesinski, et al., 2016; Behm et al., 2017). Lifting high loads (HL) requires contractions at an unintentional slow velocity enabling more time for cross-bridge formation and thus producing higher forces than when lifting low loads (Fenwick et al., 2017). Training with HL is preferential to increase maximal (1RM) or near-maximal strength (Folland and Williams, 2007; Lopez et al., 2021), and it has the potential to maximize recruitment of the motor unit pool in fewer repetitions (Lopez et al., 2021) and with less fatigue in the central nervous system than low loads (LL) training when both strength trainings are performed to momentary failure (Farrow et al., 2021). On the other hand, training with moderate loads (that is, 60%–79%) to LL (that is, 30%–59%) inflicts less stress on the joints and tendons than HL (Bohm et al., 2015) and have demonstrated the ability to increase power in a broad load spectrum (McBride et al., 2002) and the ability to apply force at high velocity in the most resistance exercises, particularly in jumping exercises in young athletes (Behm et al., 2017). Lifting LL with maximal exertion enhances high-velocity strength (McBride et al., 2002) through an increment in motor unit firing rate (Van Cutsem et al., 1998) and refinement in neural modulation at high speed (Behm and Sale, 1993). However, since high levels of maximal strength underpin power output (McQuilliam et al., 2020), and HL have demonstrated more remarkable neural adaptations than low loads (Jenkins et al., 2017), some coaches and sports scientists have employed a combination of HL and LL or the so-called contrast training to increase lower-limb power output and jump performance (Duthie et al., 2002; Marshall et al., 2021). For all these reasons, contrast training can be a valuable tool for improving the jumping ability of trampoline gymnastics athletes.

In this regard, contrast training has been defined as a workout that involves using exercises with loads in a wide range of the force-velocity profile within a sequence, that is, alternating HL and LL exercises set for set (Marshall et al., 2021). Several works have found that contrast configurations improved strength and power in young athletes (Franco-Marquez et al., 2015; Bauer et al., 2019; Fathi et al., 2019). It is suggested that contrast configuration could bring a performance enhancement in submaximal or LL high-velocity exercises when those are preceded by a biomechanically similar HL (>80% of dynamic or isometric MVC) in the same circuit set by set (Duthie et al., 2002). The physiological mechanisms that explain the chronic adaptations produced after a contrast training period remain to be determined. The prescription of HL as a conditioning activity in a contrast training sequence is not always possible with young athletes, either due to a lack of strength in the trunk muscles that limits the correct execution of the exercises or because their sport modality already implies many impacts on joints and tendons. The trampoline practice entails a high volume of impacts on joints, tendons, and ligaments, especially in the spine and lower limbs (Grapton et al., 2013). Somersault is a commonly used skill in trampoline and one of the movements that most injury produces (Lindner and Caine, 1990; Grapton et al., 2013); consequently, the athletes sustain lower limb ligament damage frequently. Trampoline gymnasts would benefit from using effective training strategies to improve jumping performance, but on the other hand, they do not impose high stress on the joints and ligaments. The use of lower limb LL blood flow restriction exercises (LL-BFRE) has proven to be an efficacious alternative to high-load exercises (HL-RE) when used alternately within the same week or alternating weekly (Hansen et al., 2020). The employment of LL-BFRE can provide a less stressful stimulus on joints and tendons with a similar level of neuromuscular adaptations (Luebbers et al., 2019). Some studies have reported that low-load resistance exercises (i.e., ∼30% 1 RM) with a high number of repetitions (12–30) (Pope et al., 2013) in combination with BFR (LL-BFRE) are effective at increasing muscle mass and strength across a wide range of populations (Loenneke et al., 2014; Loenneke et al., 2012b; Centner and Lauber, 2020; Pearson and Hussain, 2015). Interestingly, LL-BFRE and HL-RE resulted in similar levels of muscle water content (i.e., muscle swelling) (Freitas et al., 2017), mechanisms associated with post-activation performance enhancement (PAPE); however, LL-BFRE has been barely used in complex and contrast training sequences (Cleary and Cook, 2020; Doma et al., 2020). Cleary and Cook (2020) reported that in a complex strength session, both HL-RE and LL-BFRE failed to produce a PAP effect (Cleary and Cook, 2020). Nevertheless, they also pointed out that the absence of effect might be caused by an ineffective complex training protocol or other individual factors (Cleary and Cook, 2020).

Meanwhile, even though many studies have reported the effects of training with LL-BFRE on muscular strength and hypertrophy (Pearson and Hussain, 2015), the evidence of LL-BFRE’s influence on power or jumping performance is unclear. Some authors have reported that LL-BFRE or jumping exercises with blood flow restriction in the legs did not affect the power or jumping performance (Abe et al., 2005; Horiuchi et al., 2018). Conversely, Cook et al. (2014) found that three weeks of BFR training with moderate-load (i.e., 70% 1 RM) induced significant increases in strength and countermovement jump performance in young adult rugby players (Cook et al., 2014). In addition, it was found that an increase in height, flight time, and power of drop jump when LL-BFRE, bodyweight lunges with occluded legs, are performed 6–16 min before the drop jumps (Doma et al., 2020). In brief, the LL-BFRE seems to be efficient to warm-up previous to power exercises or be part of a resistance training sequence, as contrast training, to enhance posteriorly executed jumping exercises. Nevertheless, to the best of our knowledge, the effect of LL-BFRE as a conditioning activity on a contrast training sequence has not been investigated with preadolescent or adolescent athletes.

Therefore, the present work aims to study the effects of LL-BFRE, as a conditioning activity, into a contrast training scheme, on strength and power outcomes in high-level preadolescent trampoline gymnasts.

## 2 Materials and Methods

### 2.1 Participants

Fifteen preadolescent trampoline gymnasts (seven boys and eight girls) participated in the current study (age: 13.9 ± 0.4 y). All participants were high-level trampoline gymnasts, regional and national junior trampoline team members. Inclusion criteria were: 1) to play the national-level youth competitions finals; 2) to regularly train strength and conditioning, at least two sessions per week, for at least one year; and 3) to be in Tanner Stage II maturation level according to the evaluation of two certified and experienced doctors from Shanghai Sports Bureau. Legal guardians and participants provided informed consent and assent after a thorough explanation of the objectives and scope of the study, including procedures, risks, and benefits. All the procedures complied with ethical standards for research involving human participants set by the Declaration of Helsinki, and the study was approved by the Board of Research Committee of Shanghai Research Institute of Sports Science (no. 20J006, date: 30/09/2019).

### 2.2 Study Design

The present study used a matched pair design with two intervention groups and no control group. This procedure respected the “CONSORT” statement (http://www.consort-statement.org). Participants were ranked based on maximal isometric strength performance evaluated through the mid-thigh pull test (procedure detailed in testing procedures section) during the testing days pre-intervention and, once paired, were randomly allocated to one of the two experimental. One group, the HL-RE (*n* = 8, four female; age: 13.8 ± 0.5; height: 152.4 ± 7.9 cm; weight: 43.6 ± 7.2 kg; 3RM: 55.8 ± 8.4 kg), trained according to a traditional contrast sequence when the first exercise is performed with high-load resistance exercise (i.e., strength exercise), the second exercise is performed with a slow stretch-shortening cycle (SSC) exercise (i.e., power exercise) and the last one is a fast SSC exercise (i.e., plyometric exercise). The other group, the LL-BFRE (*n* = 7, four female; age: 13.9 ± 0.4 y, height: 156.7 ± 6.8 kg; weight: 43.1 ± 5.1 kg; 3RM: 56.3 ± 9.2 kg), performed identical contrast sequences and exact exercise only replacing the high-load employed by HL-RE group for low-loads with occlusive cuffs placed on the proximal area of the legs.

The participants completed a four-week familiarization period (8 sessions) before the ten-week intervention program (20 sessions of 90 min, 2 sessions per week). During familiarization sessions, participants spent one hour and a half practicing the strength, power, and plyometric exercises; meanwhile, researchers and coaches instructed the participants about the correct exercises’ technique and safety issues. Additionally, all the participants practiced the testing protocol of strength and power tests and the utilization of the BFR wraps for half an hour every week. Participants recovered at least 48 h before each testing day, before (PRE) and after (POST) the intervention.

### 2.3 Blood Flow Restriction Set Up

According to previous recommendations, blood flow restriction was applied on the legs proximally on the femur near the inguinal crease through the individualized perceived pressure method (Wilson et al., 2013). In order to be ecologic and time-efficient during training sessions, researchers established the training cuff pressure with EDGE Restriction System BFR cuffs (size: 7.62 × 74.30 cm, The Edge Mobility System, United States) matching to the score provided for the participants in a pressure visual analogic scale (VAS). The VAS ranged from 0 to 10 points, where 0 was no pressure at all, 7 was moderate pressure with no pain, and 10 was intense pressure that causes pain (Wilson et al., 2013). The target pressure was set at 7 on the VAS scale for the entire intervention. Researchers calibrated the training cuff pressure to the target perceived pressure 24 h before the first training session of every week. During each training week, the LL-BFRE group trained with the cuffs on the legs, at the pressure set during calibration session, only in the first resistance exercise of the contrast sequence (i.e., back squat and front squat). They took off the cuffs for power and plyometric exercises. Researchers registered any sign of discomfort or possible adverse effects.

### 2.4 Training Program

We measured the three-repetition maximum (3RM) test on the back and front squat to prescribe the loads for the first resistance exercise of the contrast training in both groups (for more details see Testing procedures section). After 3RM measurement, the young trampoline gymnasts performed the contrast training shown in Table 1 and the same specific trampoline and low-intensity aerobic training sessions. Both groups followed a linear periodization in the first conditioning resistance exercise (Harries et al., 2015). Recovery between exercises within the contrast sequence was one and a half minutes and 3 minutes between sets. LL-BFRE group performed the first conditioning resistance exercise with the proximal thighs occluded with the cuffs, a low-load (≤33% of 3 RM), and 3 or 4 sets of moderate effort (i.e., 10 to 12 repetitions). On the other hand, the HL-RE group performed the exact conditioning exercise but with a high load (87.5%–90% of 3RM) and 3 or 4 sets of moderate effort (4–5 repetitions). The training program aimed to improve strength and power capabilities; thus, the intensity effort of conditioning exercise was set at a moderate level (i.e., repetitions prescribed according to the 50–60% of the maximum repetitions that participants were able to lift with each conditioning during familiarization period) to avoid too much fatigue during the contrast sequence. The power and plyometric exercise were the same for both groups (Table 1).

**TABLE 1:** Training program of HL-RE and LL-BFRE groups.

		Session 1						Session 2					
		Back squat		Loaded squat jump		Drop jump from box		Front squat		CMJ with hex-barbell		Hurdle jump to box	
		Intensity	set×reps	Intensity	set×reps	Intensity	set×reps	Intensity	set×reps	Intensity	set×reps	Intensity	set×reps
week1	HL-RE	67% 3RM	3 × 10	20% BW	3 × 4	BW	3 × 4	67% 3RM	3 × 10	20 kg	3 × 4	BW	3 × 4
	LL-BFRE	25% 3RM	3 × 10					25% 3RM	3 × 10				
week2	HL-RE	75% 3RM	3 × 10	20% BW	3 × 4	BW	3 × 4	75% 3RM	3 × 10	20 kg	3 × 4	BW	3 × 4
	LL-BFRE	30% 3RM	3 × 10					30% 3RM	3 × 10				
week3	HL-RE	85% 3RM	3 × 6	30% BW	3 × 5	BW	3 × 4	85% 3RM	3 × 6	20 kg	3 × 5	BW	3 × 4
	LL-BFRE	30% 3RM	3 × 12					30% 3RM	3 × 12				
week4	HL-RE	90% 3RM	3 × 5	30% BW	3 × 4	BW	3 × 4	90% 3RM	3 × 5	20 kg	3 × 4	BW	3 × 4
	LL-BFRE	35% 3RM	3 × 12					35% 3RM	3 × 12				
week5	HL-RE	90% 3RM	4 × 4	30% BW	3 × 4	BW	3 × 4	90% 3RM	4 × 4	20 kg	3 × 4	BW	3 × 4
	LL-BFRE	35% 3RM	3 × 10					35% 3RM	3 × 10				
week6	HL-RE	85% 3RM	4 × 4	30% BW	4 × 4	BW	4 × 3	85% 3RM	4 × 4	20 kg	4 × 4	BW	4 × 3
	LL-BFRE	30% 3RM	3 × 10					30% 3RM	3 × 10				
week7	HL-RE	85% 3RM	4 × 4	30% BW	4 × 4	BW	4 × 4	85% 3RM	4 × 4	20 kg	4 × 4	BW	4 × 4
	LL-BFRE	30% 3RM	3 × 12					30% 3RM	3 × 12				
week8	HL-RE	90% 3RM	3 × 5	30% BW	3 × 4	BW	3 × 4	90% 3RM	3 × 5	20 kg	3 × 4	BW	3 × 4
	LL-BFRE	35% 3RM	3 × 12					35% 3RM	3 × 12				
week9	HL-RE	85% 3RM	4 × 4	30% BW	4 × 4	BW	4 × 3	85% 3RM	4 × 4	20 kg	4 × 4	BW	4 × 3
	LL-BFRE	30% 3RM	3 × 10					30% 3RM	3 × 10				
week10	HL-RE	90% 3RM	3 × 5	30% BW	3 × 4	BW	3 × 4	90% 3RM	3 × 5	20 kg	3 × 4	BW	3 × 4
	LL-BFRE	35% 3RM	3 × 12					35% 3RM	3 × 12				

§Exact core exercises were employed for both HL-RE and LL-BFRE, it consisted of 3 sets of 3 exercises in each session.

### 2.5 Testing Procedures

Testing sessions before and after the intervention were conducted at the same time of the day (before breakfast), the same indoor gym with a similar temperature regulated by air-conditioning (∼26°C). Evaluation sessions were under the supervision of the research team and qualified physicians and coaches. On the morning of the pre-test, two certified and experienced doctors established maturity through the Tanner scale (Marshall and Tanner, 1969; Marshall and Tanner, 1970). Then, the researchers measured the height (cm) and left and right mid-thigh girth (LTG and RTG, cm) using a measuring tape to the nearest 0.1 cm. The body mass (BM, kg) was measured by a bioimpedance scale (Inbody 270; InBody United States, Cerritos, CA, United States), and body composition was estimated from resistance obtained and through manufactured algorithms. Changes in muscle mass (MM, kg) and calculated body mass index (BMI, kg/m^2^) were calculated pre- and post-intervention.

In the afternoon, participants performed a standardized 15 min warm-up (jogging, dynamic stretching, and muscle activation) before the power and strength tests. The lower body’s power was assessed using the vertical squat jump test (SJ), the vertical countermovement jump test (CMJ), and the drop jump test from a 30 cm-height box (DJ). Furthermore, the eccentric utilization ratio (EUR) and the reactive strength index (RSI) were calculated from the jump tests (detailed below). Participants performed three maximal voluntary contractions in the isometric mid-thigh pull (IMTP). The exercise testing sequence and rest intervals between repetitions and sets for power and strength tests tried to minimize the accumulated fatigue through the evaluating session. The order within this protocol was constant: 1. SJ, 2. CMJ, 3. DJ, and 4. IMTP. The participants performed three successful repetitions, and they were blinded to the results of every repetition for ensuring the best effort in every repetition. If the last attempt was the highest, the participant performed one extra repetition to avoid possible deviations. The recovery between repetitions was 1 minute in jump tests and 2 minutes in the IMTP test. The power and strength evaluations were performed on a force platform (Kistler 9290AA, Instruments Inc., Amherst, NY, United States, sampling frequency of 1,000 Hz). The jumping height (cm) of SJ, CMJ, and DJ was automatically calculated by Mars (Version 5.0.0.0149, United States). The description of each test and the variable extracted are presented below.

#### 2.5.1 Jump Tests

##### 2.5.1.1 Squat Jump

The participants started from the static position with knees flexed to 90° and with hands on the hips (Petronijevic et al., 2018). After every repetition, researchers excluded any attempt if they detected any with countermovement by visual inspection. The repetition with the highest jumping height was used for further analysis.

##### 2.5.2.2 Counter Movement Jump

The participant stood up-right and still on the force platform for at least one second. After hearing “go” the from tester, participants counter-moved (descend up to comfortable knee flexion, as close to 90° knee flexion as possible), and then vertically jumped with maximal effort (McMahon et al., 2018). The hands were required to remain on hips throughout the whole test. Repetitions with hip or knee flexion movement in the flight phase of the jump were excluded. The repetition with the highest jumping height was employed for further analysis.

##### 2.5.2.3 Eccentric Utilization Ratio

The EUR was calculated by dividing the height (cm) reached in the CMJ by the height in the SJ. The EUR has been proposed to indicate SSC performance in various sports and during different training phases Mcguigan, 2006.

##### 2.5.2.4 Drop-Jump

The participants stood upright and still on top of the 30 cm height box with their hands on hips. After the signal, they dropped from the box and, after the contact with the platform, jumped vertically with maximal effort (Bishop et al., 2019). The researchers encouraged the athletes to jump as high and quickly as possible with minimal ground contact time. The attempts were excluded if: 1) there was hip or knee flexion during the flight phase or the contact with the platform, 2) participants jumped from the box to the platform instead of dropping, or 3) the ground contact time was longer than 250 milliseconds. The jumping height of the repetition with the highest RSI was used for further analysis.

##### 2.5.2.5 Reactive Strength Index

The RSI was calculated by dividing the height jumped by the time in contact with the ground prior to take-off during DJ (cm/seconds). This parameter has been suggested to quantify plyometric or fast SSC performance (Flanagan and Comyns, 2008).

#### 2.5.2 Maximal Isometric Mid-thigh Pull Test

IMTP was conducted following the protocol recommended by previous studies (Chavda et al., 2019). In brief, participants were instructed to adjust the body posture mimicking a power position in the clean exercise. Athletes adjusted the bar height a knee angle of about 130° and a hip angle of about 145° and made three attempts pulling the bar at 50%, 70%, and 90% of maximal effort. After 1-min rest, participants were encouraged to pull the bar as fast and hard as possible to hold the pull action for at least 4 seconds. Researchers provided solid verbal encouragement during the maximal effort attempts. Criteria of nonvalid attempts were: 1) there was visible countermovement action in the force-time curve, and 2) the presence of the peak force at the end of the pull (Chavda et al., 2019). Participants performed three IMTP attempts, and the one with the highest peak force was employed for further analysis. Relative maximal strength (IMTP-R, N*kg^−1^) was calculated as the force produced regarding the body mass.

#### 2.5.3 Three-Repetition Maximum Test

After testing sessions and aimed to prescribe training loads for the conditioning activity within the contrast sequence, participants accomplished the 3RM test in the last session of the familiarization period. A qualified strength and conditioning coach supervised the test sessions and all the training sessions. A previously recommended protocol was followed (Faigenbaum et al., 2003). Briefly, after 15-min of a standardized warm-up, young athletes completed two approaching sets of 10 and 5 repetitions with 40% and 60% of the estimated time 1RM based on their performance during the familiarization. After two recovery minutes, the load was progressively increased by 10%–20% until the athletes could no longer complete the full range of movement for more than three repetitions. The 3RM load was determined as the last weight that the athletes successfully lifted for three repetitions (i.e., muscle or technical failure) through the entire range of motion.

### 2.6 Statistical Analyses

Test-retest reliability was reported using the intraclass correlation coefficient (ICC) with 95% confident interval a single-measurement, absolute-agreement, 2-way mixed-effects model. Based on the 95% confident interval (CI) of the ICC estimate, values less than 0.5, between 0.5 and 0.75, between 0.75 and 0.9, and greater than 0.90 were interpreted as poor, moderate, good, and excellent reliability, respectively (Koo and Li, 2016). Changes within and between groups for anthropometric measures, body composition, lower-body power, and strength were analyzed using linear mixed models for repeated measures designs. Normality of the residuals was analyzed with Shapiro-Wilk test in every variable and revealed no deviations from a normal distribution. Homoscedasticity was checked by plotting the residuals-predicted value (Santos Nobre and da Motta Singer, 2007), and we found the residuals were constant across the predicted values of every variable analyzed. We employed the module GAMLj, which uses the R formulation of random effects as implemented by the lme4 R package in jamovi software (https://www.jamovi.org/). GAMLj estimates variance components with restricted (residual) maximum likelihood, which, unlike earlier maximum likelihood estimation, produces unbiased estimates of variance and covariance parameters. The intersubject factor group (LL-BFRE and HL-RE), the intrasubject factor time (PRE and POST), and the interaction (Group × Time) were set as fixed effects and participants’ intercepts were set as a random effect. F and t values and the corresponding degrees of freedom were computed. Within-subject changes were evaluated by the β coefficients and their corresponding 95% CI, representing a non-standardized effect size. Mean percentage changes (100 × [Post-Pre] × Pre^−1^) and standard deviation were calculated for all parameters. Between-group changes were evaluated by the estimated parameter with the 95% CI of the interaction between the fixed effect of the model. The alpha level was set at *p* < 0.05.

## 3 Results

### 3.1 Lower-Body Power and Strength Outcomes

**FIGURE 1:**
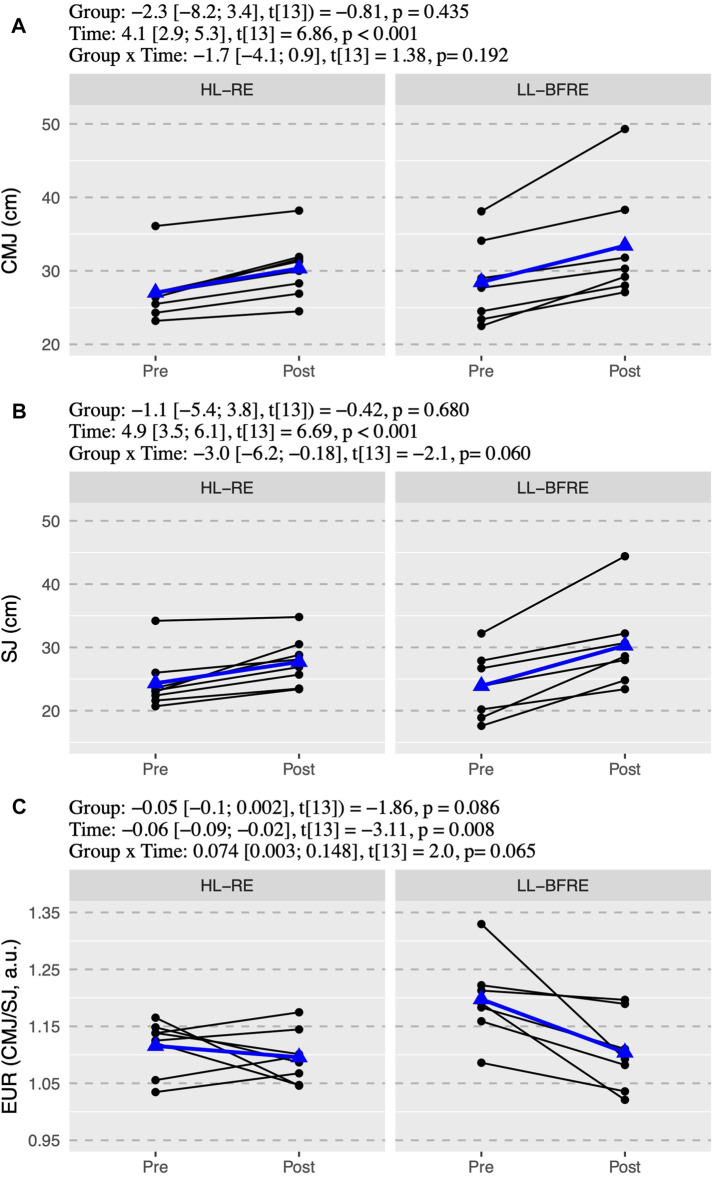
The countermovement jump **(A)**, the squat jump test **(B)**, and the eccentric utilization ratio (EUR = CMJ/SJ) **(C)** values. Black points and lines represent individual responses. Blue triangles and regression line represent the mean response. Effects of the group (HL-RE vs. LL-BFRE), time (POST vs. PRE) and interaction (HL-RE vs. LLBFRE * POST vs. PRE) are presented through beta coefficient and 95% of the confidence interval, t-value and *p*-value obtained after mixed model analysis. HL-RE: high-load exercises group; LL-BFRE: low-load blood flow restriction exercise group; CMJ: countermovement jump, SJ: squat jump test, EUR: eccentric utilization ratio.

**FIGURE 2:**
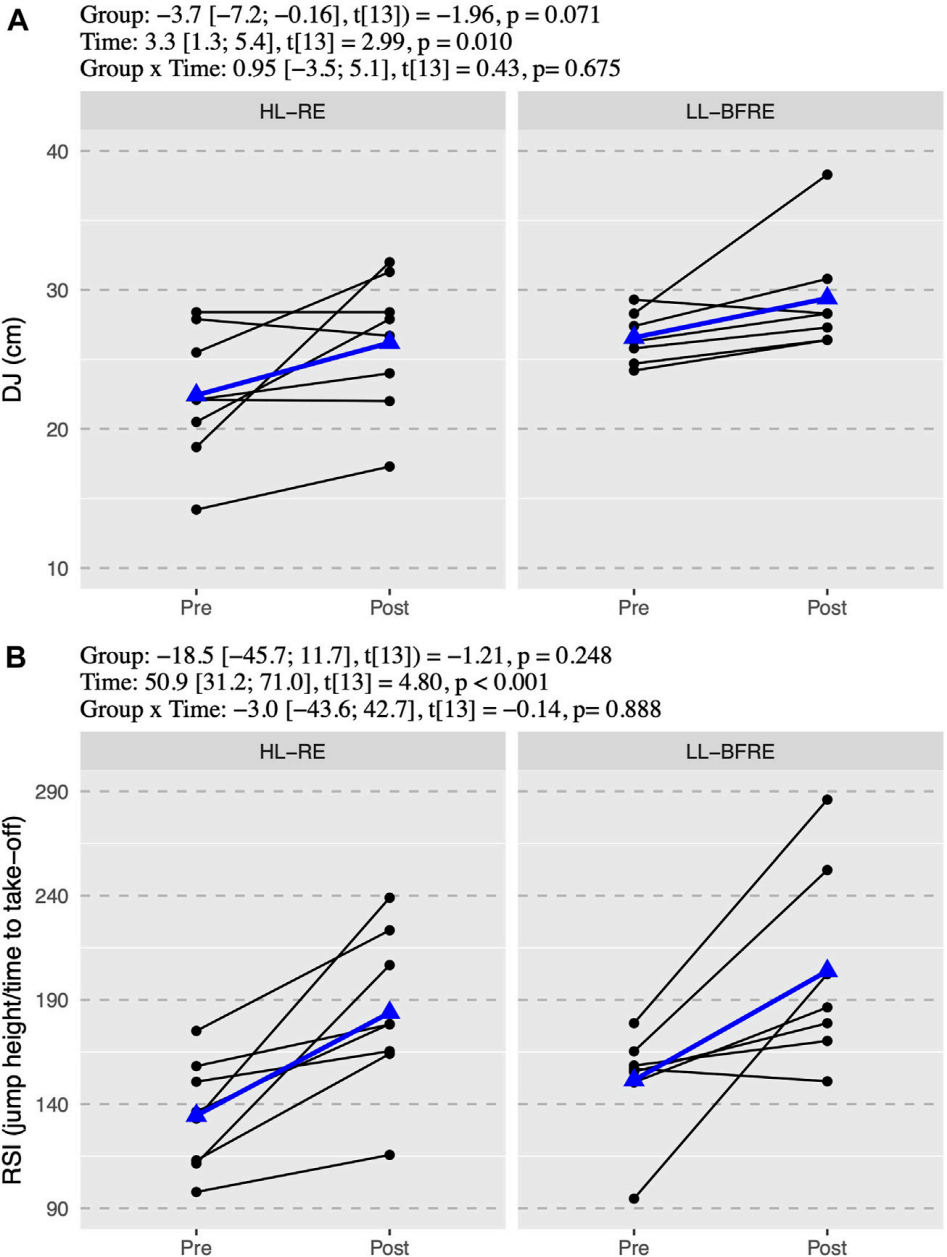
The drop-jump values **(A)** and the reactive strength index **(B)**. Black points and lines represent individual responses. Blue triangles and regression line represent the mean response. Effects of the group (HL-RE vs. LL-BFRE), time (POST vs. PRE) and interaction (HL-RE vs. LLBFRE * POST vs. PRE) are presented through beta coefficient and 95% of the confidence interval, t-value and *p*-value obtained after mixed model analysis. HL-RE: high-load exercises group; LL-BFRE: low-load blood flow restriction exercise group; DJ: drop-jump, RSI: reactive strength index calculated as jump height (cm) and ground contact time before take-off (seconds).

**FIGURE 3:**
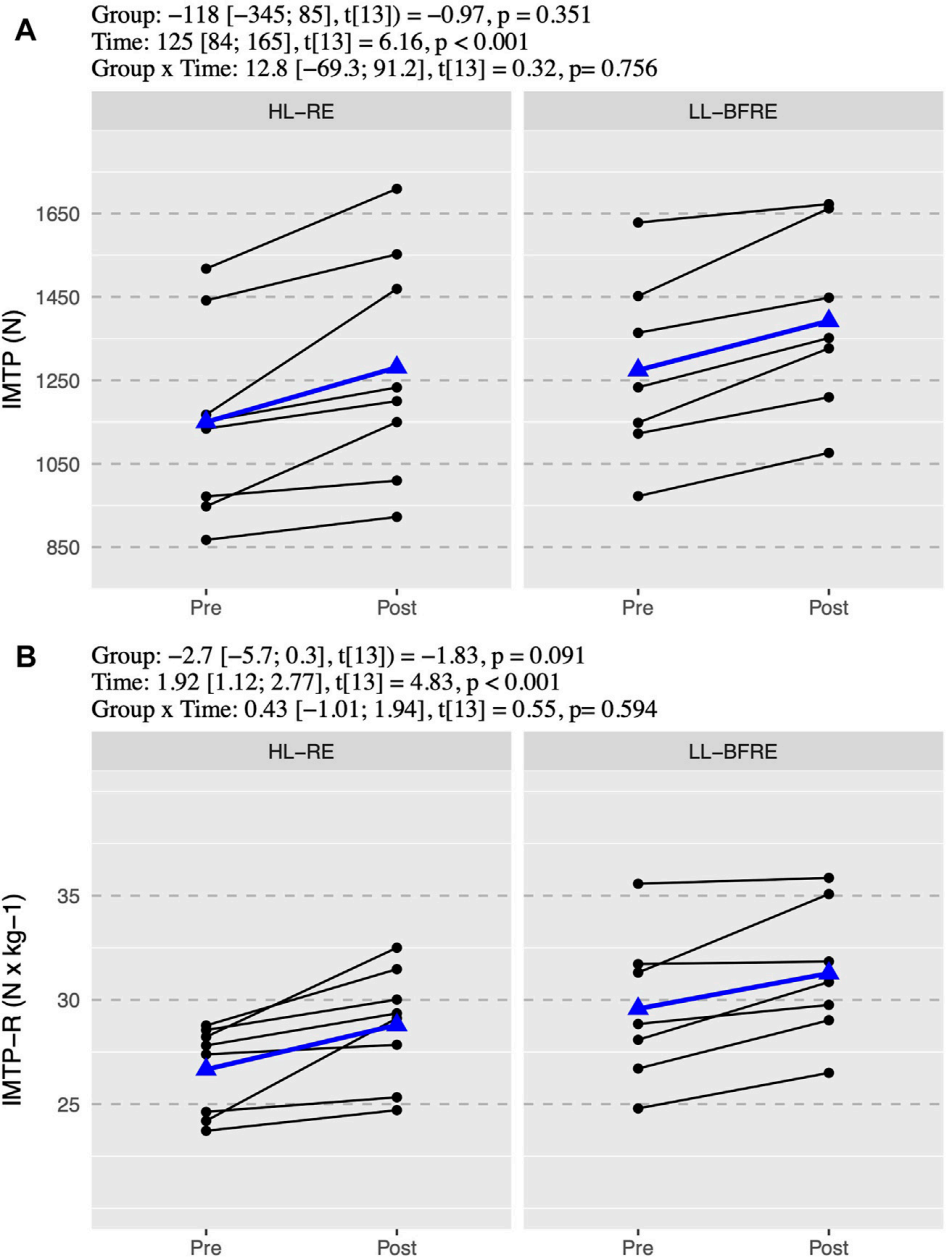
Absolute **(A)** and relative isometric mid-thigh pull **(B)**. Black points and lines represent individual responses. Blue triangles and regression line represent the mean response. Effects of the group (HL-RE vs. LL-BFRE), time (POST vs. PRE) and interaction (HL-RE vs. LLBFRE * POST vs. PRE) are presented through beta coefficient and 95% of the confidence interval, t-value and *p*-value obtained after mixed model analysis. HL-RE: high-load exercises group; LL-BFRE: low-load blood flow restriction exercise group; IMTP: isometric mid-thigh pull; IMTP-R: isometric mid-thigh pull relative to bodyweight.

The ICC with 95% CI of the variables were from “good” to “excellent” for CMJ (ICC = 0.99, 95% CI = 0.89–0.99) and DJ (ICC = 0.95, 95% CI = 0.89–0.98); and from “moderate” to “excellent” for SJ (ICC = 0.97, 95% CI = 0.65–0.99), RSI (ICC = 0.97, 95% CI = 0.67–0.99) and IMTP (ICC = 0.98, 95% CI = 0.63–0.99). There was no significant interaction (*p* > 0.05) between group x time in any power and strength outcome (Figures 1–3). However, there was an overall effect of time (*p* < 0.05) in all power and strength variables (CMJ, SJ, EUR, DJ, RSI, IMTP, and IMTP-R).

CMJ increased 12.4 ± 5.4% and 17.2 ± 8.7% for HL-RE and LL-BFRE respectively, and simple effect analysis of time within each group revealed that LL-BFRE improved CMJ a mean of 5.0 cm (95% CI = [3.1; 6.9], t_13_ = 5.64, *p* < 0.001), and HL-RE improved 3.3 cm (95% CI = [1.5; 5.1], t_13_ = 4.01, *p* = 0.001) (Figure 1). On the other hand, SJ showed a trend to significant interaction effect (*p* = 0.06) and a significant time effect (*p* < 0.001). In fact, HL-RE increased 14.6 ± 9.4% (β = 3.4 cm, 95% CI = [1.2; 5.5], t_13_ = 3.39, *p* = 0.005) and LL-BFRE estimably more, 27.6 ± 15.3% (β = 6.4 cm, 95% CI = [4.1; 8.7], t_13_ = 5.99, *p* < 0.001) (Figure 1). Differentiated changes in SJ, not experimented in CMJ, affected EUR, thus there was a tendency (*p* = 0.065) to interaction in EUR. LL-BFRE significant decreased EUR (β = −0.09 a.u., 95% CI = [−0.15; −0.04], t_13_ = −3.51, *p* = 0.004), notwithstanding HL-RE did not change EUR (β = −0.02 a.u., 95% CI = [−0.07; 0.03], t_13_ = -0.80, *p* = 0.437) (Figure 1). The mean percentage change of EUR for HL-RE was −1.7 ± 5.4% and there was the same proportion (i.e., 50%) of participants who increased and decreased their values. However, the mean percentage change of EUR for LL-BFRE was −7.6 ± 6.1% and all the participants in that group decreased their EUR (Figure 1).

Outcomes in DJ showed a high variability of responses within groups (ICC random intercept = 0.482). DJ did not show interaction effect (*p* = 0.675), but it showed a time effect that analyzed within each group revealed a 3.8 cm DJ improvement in HL-RE (95% CI = [0.5; 7.0], t_13_ = 2.50, *p* = 0.026), and not changes for LL-BFRE (β = 2.8 cm, 95% CI = [-0.7; 6.3], t_13_ = 1.76, *p* = 0.103) (Figure 2). Despite the different evolution in the DJ, the RSI improved in both groups 38.7 ± 29.3% (β = 49.4, 95% CI = [18.1; 80.7], t_13_ = 3.41, *p* = 0.005) in HL-RE and 38.4 ± 40.6% (β = 52.4, 95% CI = [18.9; 85.9], t_13_ = 3.38, *p* = 0.005) in LL-BFRE (Figure 2).

We observed a similar mean percentage improvement in IMTP for HL-RE (11.3 ± 8.0%) and LL-BFRE (9.6 ± 4.5%). Moreover, simple effects analysis showed that both groups improved IMTP (HL-RE: β = 131 N, 95% CI = [71; 191], t_13_ = 4.74, *p* < 0.001; and LL-BFRE: β = 118 N, 95% CI = [54; 182], t_13_ = 4.00, *p* = 0.002). The IMTP-R increased similarly in both groups (HL-RE: β = 2.1 N/kg, 95% CI = [0.9; 3.3], t_13_ = 3.93, *p* = 0.002; and LL-BFRE: β = 1.7 N/kg, 95% CI = [0.5; 2.9], t_13_ = 2.93, *p* = 0.012) (Figure 3).

### 3.2 Anthropometric Measures and Muscle Mass Changes

Changes in anthropometric measures and body composition are presented in Table 2. There was a significant interaction (group x time) in muscle mass (MM) (β = 0.57 kg, 95% CI = [0.15; 0.98], t_13_ = 2.67, *p* = 0.019), revealing that participants in LL-BFRE increased their muscle mass (6.6 ± 3.1%) compared to HL-RE (3.6 ± 2.0%). The fixed effect omnibus test did not reveal any group or interaction effect in the anthropometric variables. However, there was a time effect on all the variables (*p* < 0.05). Group simple effects within PRE revealed no significant differences between groups, which ruled out group allocation or initial bias. The simple effects of time within each group are shown in Table 2.

**TABLE 2:** Changes in body composition of low-load blood flow restriction exercise group (LL-BFRE) and high-load exercise group (HL-RE).

	LL-BFRE (*n* = 7)		POST vs. PRE		HL-RE (*n* = 8)		POST vs. PRE		LL-BFRE vs. HL-RE		Group×Time Interaction	
	PRE	POST	β [95% CI]	*p*-value	PRE	POST	β [95% CI]	*p*-value	β [95% CI]	*p*-value	β [95% CI]	*p*-value
Weight (kg)	43 ± 7	45 ± 7	1.4 (0.8; 2.1)	<0.001***	43 ± 7	44 ± 7	1.3 (0.7; 1.9)	<0.001***	0.04 (−6.6; 6.7)	0.990	0.14 (−0.7; 0.9)	0.730
Height (cm)	157 ± 7	158 ± 7	1.4 (1.0; 1.8)	<0.001***	152 ± 8	153 ± 8	1.1 (0.7; 1.5)	<0.001***	4.51 (−3.1; 12.1)	0.264	0.36 (−0.2; 0.9)	0.199
BMI (kg/m^2^)	18 ± 1	18 ± 1	0.3 (0.0; 0.5)	0.049*	18 ± 1	19 ± 1	0.3 (0.1; 0.5)	0.022*	−0.96 (−2.2; 0.3)	0.164	−0.03 (−0.4; 0.3)	0.851
MM (kg)	20 ± 3	21 ± 3	1.2 (0.9; 1.6)	<0.001***	19 ± 3	20 ± 3	0.7 (0.4; 1.0)	<0.001***	0.57 (−2.8; 3.9)	0.742	0.57 (0.15; 0.98)	0.019*
LTG (cm)	43 ± 4	45 ± 4	2.0 (1.3; 2.7)	<0.001***	43 ± 4	45 ± 4	1.9 (1.2; 2.6)	<0.001***	−0.63 (−4.1; 2.9)	0.728	0.09 (−0.8; 1.0)	0.851
RTG (cm)	43 ± 8	45 ± 43	1.0 (0.3; 1.8)	0.011*	44 ± 4	45 ± 4	1.2 (0.5; 1.8)	0.003**	−0.65 (−4.4; 3.1)	0.737	−0.14 (−1.1; 0.8)	0.778

Data are estimated marginal means ± standard deviation. PRE, vs. POST, is a simple effects analysis within each group. 95% CI: confidence interval at 95%; LL-BFRE: low-load blood flow restriction exercises group; HL-RE, high-load exercises group; PRE, preintervention assessment; POST, postintervention assessment; BMI, body mass index; MM, muscle mass; LTG, left mid-thigh girth; RTG, right mid-thigh girth. Significance: *p < 0.05, **p < 0.01, ***p < 0.001.

## 4 Discussion

We found that preadolescent trampoline gymnasts increased their jump height (CMJ, SJ) and strength capabilities (IMTP) regardless of employing HL or blood flow restricted LL as conditioning activity within a contrast training strength program performed two days per week for ten weeks. These results suggest the potential usefulness of BFR with low loads as a conditioning activity in a contrast training sequence in preadolescents athletes. However, our results must be interpreted with caution since we could not have a group that only trained specific trampoline sessions and thus compare the magnitude of change between the two interventions group with a control group. Participants’ allocation was based on the rank obtained from the maximal isometric mid-thigh pull test in the pre-intervention to prevent participants’ force level from influencing the present study’s independent variable (i.e., the type of conditioning activity). Moreover, the groups did not significantly differ in any jump measures (i.e., CMJ, SJ, DJ) in the PRE, meaning that post-training differences could not be attributed to unequal group composition or pre-experimental biases.

The present work is the first investigation to study the effects of contrast-type resistance exercise training on the performance of high-level early adolescent trampoline gymnasts. The increments observed CMJ (HL-RE: 12.4 ± 5.4% and LL-BFRE: 17.2 ± 8.7%) and SJ (HL-RE: 14.6 ± 9.4% and LL-BFRE: 27.6 ± 15.3%) in both groups were much higher than those reported in the literature for pubertal volleyball players when trained with plyometrics (CMJ: 3.4% and SJ: 4.1%) or combining resistance and plyometric exercises (CMJ: 6.3% and SJ: 7.1%) (Fathi et al., 2019). In the last experiment, authors implemented a control group that continued their regular volleyball training, and they reported no changes in CMJ and SJ after 16-weeks of regular training (Fathi et al., 2019). A higher volume of jumps is performed during trampoline gymnastics sessions than in volleyball training, and likely a control group would show an improvement in those jumps. However, the magnitude of improvement reached in the present study after only ten weeks of two different contrast training configurations in pre-pubertal athletes warrants more studies that might highlight the contribution of that training strategy to increasing jump capabilities in young athletes. It has been signaled that jump trainability is mediated by biological maturation (Moran et al., 2017), that conclusion is derived from a meta-analysis of plyometric controlled trials in young individuals where authors indicated that adaptative responses were higher between the mean ages of 10 and 12.99, and between 16 and 18 years, than the mean ages of 13 and 15.99 despite greater exposure (Moran et al., 2017). Surprisingly, the athletes from our study were ∽14 years old what becomes the results obtained in the present study even more relevant because we found significant improvements in athletes that were within a period of lowered response to maximize performance.

Trampoline athletes in LL-BFRE improved more SJ than CMJ (27.6 ± 15.3% vs. 17.2 ± 8.7%, respectively), contrasting to HL-RE that experimented a similar improvement in both exercises (14.6 ± 9.4% vs. 12.4 ± 5.4%, SJ and CMJ respectively). SJ and CMJ performance depends on common factors related to changes in muscle structure (Moran et al., 2017) and neural drive to muscles and on specific factors related to the ability to manage SSC (Kozinc et al., 2021). We believe that even the athletes in LL-BFRE reduced their EUR, more increment in CMJ in LL-BFRE might show that LL-BFRE did not weaken their capability to use the eccentric phase but simply increased more SJ. We also consider that one possible reason for this outcome might be the higher increment in muscle mass in the LL-BFRE group. The muscle mass increment could have contributed to the improvement of SJ and to a lesser extend in CMJ (Van Hooren and Zolotarjova, 2017; Radnor et al., 2018). Jumps with a previous countermovement require mastery stretching shortening cycle (SSC) characterized by an eccentric “stretching” action prior to a subsequent concentric “shortening” action (Nicol et al., 2006). Performance during an SSC is attributed to muscle pre-activation, the stretch reflex, and the release of stored passive-elastic energy in the muscle-tendinous tissue (Groeber et al., 2021). An augmentation in muscle mass is usually produced by an increment in contractile and no-contractile tissue. Differences in the proportion of contractile and no-contractile augmentation because of the type of conditioning activity within the contrast sequence might be the reason for the differences in the increment of performance between SJ and CMJ.

As aforementioned, trampoline athletes in LL-BFRE gained more muscle mass than those in HL-RE (6.6 ± 3.1% vs.3.6 ± 2.0%, respectively). The result of our study is consistent with the evidence from other previous studies employing BFR with young adult populations (Loenneke et al., 2012b). Furthermore, it has been observed that LL-BFR training stimulated physiological factors associated with skeletal muscle hypertrophy conversely to training without BFR with equal relative loads (20% 1RM) or even higher (50% 1RM) (Haddock et al., 2020). Since LL-BFRE performed more repetitions than HL-RE and athletes lifted at moderate effort intensity, we suggest that these circumstances might be responsible for the muscle mass changes. It has to be highlighted that the maturity process during the intervention period could have influenced the results. Food intake was not controlled during the study; however, athletes made their meals in the dining room inside the training center; thus, it is not expected that there have been considerable differences in the quality and quantity of the food intake.

On the other hand, the current study demonstrated that a contrast training program using low-loads with BFR as a conditioning activity might provide an effective and equivalent positive influence on maximal strength compared to using high-loads for preadolescents athletes. Our results agree with the evidence from other previous studies employing low load BFR with other populations (Loenneke et al., 2012b; Yamanaka et al., 2012; Luebbers et al., 2019; Hansen et al., 2020) and high school adolescent weightlifters (Mohamed et al., 2017; Luebbers et al., 2019). No side-effects (hazards or unbearable discomfort) were reported during the ten weeks of training, which implicates that LL-BFRE integrated into a contrast training sequence is effective and safe for young athletes’ strength development. Potential mechanisms of LL-BFRE training for young athletes was not within the scope of the current study, but previous research has suggested that the increased hormone level (such as plasma concentration of growth hormone) (Manini and Clark, 2009), fast-twitch fibers recruitment, and muscle cell swelling (Loenneke et al., 2012a) might be the factors for improvement of maximal strength in LL-BFRE.

The contrast training sequence is characterized by high-load resistant strength exercises followed by lighter-loads with similar movement pattern power exercises and plyometric exercises (Marshall et al., 2021) that try to cause a PAPE effect within the sets and between them. Initially, some authors suggested that the acute physiological effects linked to post-activation potentiation (PAP) (e.g., improvement of the sensitivity of the Ca^2+^ released by the sarcoplasmic reticulum due to phosphorylation of the regulatory light chain of myosin) caused by a precedent conditioning activity is the cause of improved performance in high-speed strength or power exercises during a session (Sale, 2002). The repetition of contrast training configurations can achieve a long-term improvement in the performance of activities such as jumping, throwing, or all those that depend on high values of rate of force development (Sale, 2002; Tillin and Bishop, 2009). However, other authors have exposed their concerns about PAP causing an improvement in force production after conditioning activity because the specific physiological mechanism of PAP is based on the contractile response after a conditioning activity that could or not contribute to the posterior performance enhancement (Cuenca-Fernandez et al., 2017; Boullosa et al., 2020). Conversely, it has been indicated that highly probable that other physiological effects such as changes in muscle temperature, the water content of muscles and cells, and muscle activation may be responsible, at least in part, for the improvement in voluntary strength and power (Blazevich and Babault, 2019) after a conditioning activity, the so-called PAPE (Cuenca-Fernandez et al., 2017). These physiologic mechanisms related with PAPE may have been responsible to performance increases in HL-RE and LL-BFRE contrast training sequences in our study. Cleary and Cook (2020) studied the acute effects of HL-RE and LL-BFRE in a complex training sequence in college-aged men, and they did not find an increase in the subsequent vertical jumps’ height. Conversely, our results support that HL-RE and LL-BFRE could provide a performance enhancement in jump and strength capabilities when employed chronically (i.e., ten weeks) within a contrast training sequence in pre-adolescents. If PAPE effects are responsible for improving the performance observed in contrast training with HL or LL-BFR as conditioning activity, it should be clarified in future studies. Furthermore, it has to be highlighted that LL-BFRE group trained their lower limb extension pattern at execution speeds closer to those sport-specific. As stated almost three decades ago, velocity specificity of resistance training has demonstrated that the greatest strength gains occur at or near the training velocity (Behm and Sale, 1993). Therefore, the LL-BFRE group was training at a higher velocity than HL-RE, trampoline-specific performance in longer training interventions. The results of our study should be contrasted with future designs that analyze structural and neural chronic adaptations of contrast training using LL-BFRE in young athletes’ power performance.

Moreover, our study did not find an interaction effect on reactive, explosive strength (DJ), but simple effects showed an increment in the HL-RE and no changes in LL-BFRE. As shown in Figure 2, the responses to the DJ test in HL-RE presented high variability between participants. The reason that could explain high variability in the DJ responses of the HL-RE group may be that high loads training presented specific difficulties related to the capacity to place the load on the gymnasts’ back and subsequent demands for their core strength and stability. Core weakness limits load progression and applying force efficiently when performing strength exercises like back or front squat. Nevertheless, high loads contribute to stiff muscle-tendon structures (Fenwick et al., 2017), which can explain the magnitude of response experimented by some athletes.

### 4.1 Limitations

Our study was not without limitations. Small sample size and the absence of a control group training only specific sessions must be addressed in future investigations. Furthermore, a study design with bigger samples with higher representation by sex is warranted to understand better sex differences in adaptations to LL-BFRE in power and strength training. Lastly, electrophysiologic measures to analyze motor unit recruitment and firing rate and changes in tendon-muscle architecture can give valuable information about the origin of the adaptations to training with LL-BFRE as a conditioning activity within a contrast training sequence.

## 5 Practical Applications

Low-intensity resistance training with BFR could be implemented into an integrated strength training program with a combination of power or plyometric exercise to effectively improve the strength and jump height performance in lower limbs in preadolescent athletic populations. Our study has provided valuable implications for coaches working with elite young athletes, especially when using high loads in a contrast training structure is not possible.

## 6 Conclusion

Implementing LL-BFRE in place of HL-RE in a contrast training structure is safe and might be equally effective in improving lower-body strength and power in preadolescent trampoline gymnasts.

## Data Availability

The datasets presented in this study can be found in online repositories. The names of the repository/repositories and accession number(s) can be found below: https://osf.io/789gy Name of repository/repositories: OSF.

## Author’s Note

The study was conducted at Shanghai’s Chongming Training Base of Professional Sports.
